# Early and Chronic Postnatal Depression, Maternal Sensitivity to Non‐Distress and Infant Neurodevelopmental Outcomes in an Indian Birth Cohort

**DOI:** 10.1111/infa.70103

**Published:** 2026-06-25

**Authors:** Matthew Bluett‐Duncan, Thomas Kishore, Veena Satyanarayana, Supraja TA, Laura Bozicevic, Chaithra Holla, Nicky Wright, Ellie Preece, Andrew Pickles, Prabha Chandra, Helen Sharp

**Affiliations:** ^1^ Division of Neuroscience The University of Manchester Manchester UK; ^2^ Department of Clinical Psychology National Institute of Mental Health and Neurosciences Bengaluru India; ^3^ Department of Psychiatric Social Work National Institute of Mental Health and Neurosciences Bangalore India; ^4^ Department of Primary Care and Mental Health University of Liverpool Liverpool UK; ^5^ School of Psychology, Faculty of Health and Education Manchester Metropolitan University Manchester UK; ^6^ Institute of Psychiatry, Psychology and Neuroscience Kings College London London UK; ^7^ Department of Psychiatry National Institute of Mental Health and Neurosciences Bengaluru India

## Abstract

This study examined whether findings from High‐Income Countries (HIC) suggesting that (1) maternal postnatal depression (PND) is associated with poorer infant neurodevelopmental outcomes, (2) maternal sensitivity to non‐distress (SND) is associated with improved outcomes, (3) SND may buffer the effect of PND, are present in India. Participants were from the Bangalore Child Health and Development Study (BCHADS), a prospective longitudinal cohort study starting in pregnancy (*n* = 337). Mothers self‐reported depression symptoms on the Edinburgh Postnatal Depression Scale; variables reflecting early PND (8 weeks) and chronic PND (factor score created from 8 weeks, 6, 12, 24 months) were examined. SND was coded from a semi‐structured play observation at age 6 or 12 months. Infant neurodevelopment was assessed at 24 months using the Bayley Scales of Infant Development. Early PND, chronic PND or maternal SND were not associated with infant cognitive or language development in the whole sample, but in boys increased maternal SND was associated with improved language development. SND did not moderate associations between maternal PND and outcomes. A number of factors, such as parity, birthweight by gestational age, and the presence of an alternate caregiver, demonstrated small but significant sex‐specific associations with neurodevelopment in exploratory analyses. Maternal SND was associated with improved language outcomes after accounting for multiple risks, but only in boys. Future work should examine whether broader aspects of the nurturing environment contribute to neurodevelopment in a sex‐specific manner.

## 1 Introduction

### 1.1 Background

There is widescale recognition that the time from conception to 2 years of age is extremely important for development and represents a time of heightened sensitivity to external influences (Black et al. 2017). Infant neurodevelopment during this period is typically conceptualized and assessed in terms of developmental milestones across the domains of cognitive, motor, communicative and social‐emotional development (Bayley 2006). This early period may confer a developmental advantage or vulnerability depending on environmental exposures (Teicher and Samson 2016). A common adversity experienced during this period is maternal postnatal depression, with a recent meta‐analysis reporting a prevalence of 19% in India across all measurement types, and 24% using screening instruments (Upadhyay et al. 2017). Postnatal depression constitutes a potentially significant episodic or chronic adversity that may compromise nurturing care and thus impair development (Fagan et al. 2007; Hoffman et al. 2017; Rose, Feldman, and Jankowski 2012; Rose, Feldman, Jankowski, and Van Rossem 2012; Britto et al. 2017). While the evidence base in low‐ and middle‐income countries (LMICs) regarding this association has grown considerably in recent years, further work is required to understand the association in these contexts (Sridhar et al. 2025; Bluett‐Duncan et al. 2021).

### 1.2 Early Postnatal Versus Chronic Exposure in HICs

In high‐income settings, exposure to maternal depression in infancy is conceptualized either as an early postnatal “timing” effect (birth to 12 months) or as chronic exposure across infancy. While this definition of the early postnatal period is considerably longer than standard definitions of the postnatal period, which typically range from 6 weeks to 6 months, this is how it has been conceptualized in the literature, and so is utilized here for consistency (Austin 2014; Gelaye et al. 2016).

While there is some evidence for a timing effect of maternal depression in HICs, findings are inconsistent. A number of studies have found a significant association between depression during the first postnatal year and infant neurodevelopment (Koutra et al. 2013; Lyons‐Ruth et al. 1986; Kawai et al. 2017; Murray 1992; Smith‐Nielsen et al. 2016; Whiffen and Gotlib 1989; Conroy et al. 2012; Perra et al. 2015; Bornstein et al. 2012) but a similar number of studies did not (Hanley et al. 2013; Keim et al. 2011; Kiernan and Huerta 2008; Piteo et al. 2012; Stanley et al. 2004; Stein et al. 2008; Kaplan et al. 2014). There is, however, much stronger evidence to suggest that chronic exposure to depression over a longer period during infancy can impair neurodevelopment (Brennan et al. 2000; NICHD Early Child Care Research Network 1999; Azak 2012; Cornish et al. 2005; Park et al. 2018; Milgrom et al. 2004; Sutter‐Dallay et al. 2011).

### 1.3 Existing Findings From LMIC Literature

Recent studies exploring the association between perinatal depression and infant neurodevelopment in India have produced inconsistent results. Koshy et al. (2021) reported that maternal depression in the postnatal period was associated with poorer language, but not cognitive, development measured at 24 months using the Bayley Scales of Infant Development—Third Edition (BSID‐III) in a sample recruited from a low‐income urban area of Vellore (*n* = 228). Roy et al. (2022) found that antenatal, but not postnatal, depression was associated with mental development at 12 months using the Developmental Assessment Scale in Indian Infants (DASII; *n* = 174). Using the BSID‐III, Thomas et al. (2020) report a univariate association between antenatal depression and language development at 30 months (*n* = 203) but this was lost after adjustment. Finally, an older study from rural Goa reported a significant association between postnatal depression and cognitive development at 6 months (Patel et al. 2003). More broadly, two systematic reviews of LMIC studies concluded that findings remain inconsistent and methodologically limited, with five studies reporting significant associations and eight studies reporting none (Sridhar et al. 2025; Bluett‐Duncan et al. 2021).

### 1.4 Maternal Sensitivity and Infant Neurodevelopment

Nurturing care is key for promoting optimal infant development, particularly under adverse socioeconomic conditions, fundamental to which is the concept of maternal sensitivity within the mother‐child relationship (Britto et al. 2017). The ability of the mother to perceive and respond contingently and appropriately to child's cues is thought to promote both language and cognitive development (Rocha et al. 2020), through promoting attachment security and the development of the strategies needed for an infant to develop emotional and behavioral regulation skills (Grant et al. 2010). Sensitivity to non‐distress (e.g., smiles and vocalizations occurring during play) (SND), in particular, has been demonstrated to be associated with child cognitive outcomes (Bozicevic et al. 2025). Conversely, disruptions in the caregiving relationship, such as depressive symptoms, may heighten developmental risk by amplifying infants' exposure to early life stress, undermining a mother's capacity to respond to her infant's social and emotional needs through appropriate scaffolding, and impeding the establishment of early physiological regulatory processes (Goodman 2007).

Research indicates that sensitive caregiving can be compromised by maternal depression (Bernard et al. 2018), and that impaired SND may be a key pathway through which maternal depression exerts its effect on infant neurodevelopment (NICHD Early Child Care Research Network 1999; Milgrom et al. 2004; Gueron‐Sela et al. 2018) with some evidence suggesting that male infants are more at risk from maternal depression and impaired maternal sensitivity (Azak 2012; Sharp et al. 1995; Murray et al. 1996, 2010). However, although there is a strong rationale for viewing maternal sensitivity as a pan‐cultural concept (Mesman et al. 2018), there is very little research examining its association with infant neurodevelopment in LMICs.

Most studies of maternal depression and maternal sensitivity do not consider sex‐specific associations. However, there is an indication from HIC studies that maternal depression may be more strongly associated with cognitive outcomes in boys (Grace et al. 2003). Additionally, evidence from HICs generally indicates that girls show better language development than boys (Riva 2023). LMIC data, including a large multi‐country study in the Asia‐Pacific region, similarly show girls scoring higher on composite developmental indices (Weber et al. 2017). These sex differences in neurodevelopment could indicate that there are sex‐specific risk and protective factors impacting development. In India, gender‐bias favoring boys (Barcellos et al. 2014) and gender‐discrimination in allocation of resources (Mishra et al. 2004) underscore the importance of examining sex‐specific effects.

### 1.5 Aims of the Current Study

The aim of this study is to advance the literature regarding the relationship between maternal postnatal depression, maternal SND, and infant neurodevelopment, within the general LMIC context and, more specifically, within India. Neurodevelopment was assessed at 2 years as this represents a period of increased developmental stability and better predictive utility for later outcomes (Black et al. 2017). Given the evidence reported in HIC literature that prompted this research, the study examined the following hypotheses in both the full sample and separately in males and females:

H 1 Infants exposed to early postnatal depression and to chronic postnatal depression will show poorer neurodevelopmental outcomes compared with non‐exposed infants.

H 2 Higher levels of maternal SND will be associated with better infant neurodevelopmental outcomes.

H 3 Maternal SND will moderate the association between maternal depression and infant neurodevelopment, such that the negative impact of maternal depression will be stronger when maternal SND is lower.

## 2 Method

### 2.1 Study Design

Participants were mothers and infants taking part in the Bangalore Child Health and Development Study (BCHADS), a prospective longitudinal cohort study starting in pregnancy and designed to study early risk and protective factors for childhood mental health throughout pregnancy and infancy, in India.

### 2.2 Study Setting

Bangalore is divided into nine zones, which are further subdivided into a total of 198 wards. Permission was obtained from the local municipality, “Bruhat Bangalore Mahanagara Palike” (BBMP), for the recruitment of participants who were registered at the Banashakari Urban Primary Health Center (UPHC), which serves as the Government Referral Hospital in the south zone of the Bangalore Urban District. This site was chosen because it caters to women from low resource settings and is also reachable within an hour from the main study site.

### 2.3 Recruitment Strategy

Recruitment to the study took place between July 2014 and November 2016. All participants were recruited from a Government Referral Hospital—Urban Primary Health Centers (UPHCs) at Banashankari, N.R. Colony and at Siddaiah Road. A consecutive sample of expectant mothers were approached by research assistants when attending appointments at three community antenatal clinics during their first or second trimester and screened for inclusion in the study.

Women who had a diagnosis of either psychosis or a bipolar disorder, who were identified to have major health complications during the current pregnancy, who reported harmful use of alcohol or other psychoactive substances, who did not speak the language for assessment (Kannada), or did not plan to reside in Bangalore were excluded from the cohort.

Informed consent was sought and women who consented to be part of the study were recruited to the cohort. Out of the 1048 women approached, 909 women consented to participate, 698 during trimester 1 and 211 during trimester 2. 84 of these mothers were excluded at later time‐points in the study: 44 because the pregnancy was high risk or had a poor outcome (e.g., miscarriage, medical termination, intrauterine death), five because of still birth, 24 because of neonatal death (under 1 month), six because of infant death (under 1 year), and five because infants were twins. Therefore, 825 women remained eligible for postnatal follow‐up.

### 2.4 Procedure

This report includes data from a subset of measures in the wider assessment battery. Participants were assessed in the antenatal period during trimester one (T1: maternal questionnaire), trimester 2 (T2: maternal questionnaire), and trimester 3 (T3: maternal questionnaire).

After delivery (T4) women received a telephone call from the research assistants to collect data about the pregnancy outcomes. Additional information regarding birth outcomes was retrieved from routine medical records.

Postnatal assessments took place at 6–12 weeks postpartum (T5: maternal questionnaire), 6 months postpartum (T6: maternal questionnaire, mother‐child observation), 12 months postpartum (T8: maternal questionnaire, mother‐child observation), 24 months postpartum (T9: maternal questionnaire, infant neurodevelopment). Postnatal follow‐ups took place at the NIMHANS Center for Wellbeing or at home.

### 2.5 Sample

Of the 825 families who were eligible for follow‐up from birth 742 families (89.9%) gave data at least once postpartum. This report contains data from women who provided depression data for at least 2 postnatal assessments and provided data for each of the relevant covariates (see Section 2.6) (*n* = 337). This restricted sample was broadly similar to the full cohort that provided data for at least one postnatal assessment (Table 1).

**TABLE 1:** Comparison of the full cohort and analyzed sample on key study variables.

Measure	Full cohort (*n* = 742)	Analyzed sample (*n* = 337)	Comparison *p* value
Maternal age (M, SD)	23.04 (3.45)	23.21 (3.99)	0.057
Education (up to secondary)	69.7%	70.0%	0.342
SES (BPL/LSES)	56.5%	51.6%	0.289
Religion			0.358
Hindu	84.1%	85.3%	
Muslim	15.2%	14.1%	
Christian	0.7%	0.6%	
Parity (primiparous)	45.5%	42.4%	0.053
Alternate caregiver (present)	76.0%	89.3%	0.311
Child			
Gender (male)	52.0%	51.6%	0.159
Term (37–42 weeks)	80.4%	87.2%	0.220
Birth weight—kg (M, SD)	2.90 (0.53)	2.87 (0.49)	0.084

*Note: p* values calculated using *t*‐tests for continuous variables and *χ*2 for categorical variables.

### 2.6 Measures

#### 2.6.1 Maternal Depression

Maternal depression was assessed at each phase using a Kannada version of the Edinburgh Postnatal Depression Scale (EPDS; J. L. Cox et al. 1987) that was translated following World Health Organization guidelines. The EPDS is a 10‐item Likert scale self‐report instrument designed to detect postnatal depression by focusing on the cognitive and affective aspects of depression rather than somatic symptoms. It has been widely used and validated in a number of settings (Eberhard‐Gran et al. 2001). A cut‐off of ≥ 13 is routinely used in western settings. However, when applied in different cultural contexts wide variation in psychometric properties has been reported, indicating that valid thresholds may vary considerably between contexts (Gibson et al. 2009). We have demonstrated in previous work within this cohort that Indian participants are likely to under‐report symptoms of maternal depression relative to a UK‐based cohort (Bluett‐Duncan et al. 2024). This may be due to the increased tendency for individuals from Southeast Asian societies to describe mental health difficulties in somatic, rather than cognitive or emotional, terms or due to the insensitivity of the EPDS to measure long‐term depressive symptomology in the context of increased socio‐economic adversity (Jatchavala and Sidi 2025; Tran et al. 2011).

In view of this, the Kannada EPDS was validated against diagnoses of major depressive disorder. Data suggested the existence of distinct reporting styles between the antenatal and immediate postnatal period (8 weeks) and later in infancy (6–24 months), so two separate cut‐off scores were validated using independent samples. A cut‐off of ≥ 3 (sensitivity = 62.5%, specificity = 73.9%) was selected for phases T1–T5 and a cut‐off of ≥ 10 (sensitivity = 95.2%, specificity = 83.1%) was selected for phases T6–T9. The full details of the ROC analyses are reported in Supporting Information S1: Appendix 1. This is similar to thresholds reported by a validation study performed with the EPDS in Vietnam (Tran et al. 2011).

Early postnatal depression was measured using the T5 EPDS data only. Those scoring above the cut‐off were identified as depressed and the data was converted into a binary variable (0 = non‐depressed, 1 = depressed) for use in analysis. The chronic depression exposure was measured by creating a factor score from binary EPDS data at T5, T6, T8 and T9, using the relevant cut‐off at each timepoint (see Section 2.7).

#### 2.6.2 Maternal Sensitivity

Maternal sensitivity was assessed using a standard 15‐min semi‐structured play‐based procedure adapted from the NICHD mother‐infant interaction scales at either the 6‐ or 12‐month assessment. These time‐points were selected as they represent a developmentally appropriate timepoint for assessing the impact of maternal sensitivity on child development (NICHD Early Child Care Research Network 1999). Interactions were coded using the “Qualitative Ratings for Parent‐Child Interaction at 3–15 Months of Age” (M. Cox and Crnic 2003). During the first 7‐min, mothers were asked to play with the infants using a familiar toy or object. For the remaining 8‐min, mothers were given a standard set of toys to play with. Toys for the standard set were selected to be broadly parallel to the standard set used in western settings but familiar to mothers and infants in India. Assessments were recorded using two static video cameras placed at different angles to capture facial expressions and interactions as the dyad moved around the room. A play mat was positioned in the optimal field of view for the cameras and mothers were asked to remain on it where possible. When assessments were conducted at home, researchers recorded assessments using a single portable video camera and tripod.

The NICHD coding scheme provides a wide range of observer‐rated dimensions of caregiving. In this study, only the SND rating was used as an indicator of maternal sensitivity. SND captured the extent to which a mother noticed and responded promptly and appropriately to the social gestures, expressions, and signals of the infant, and how well timed, paced and child centered her responses were. Each scale is coded on a 5‐point global impression scale from 1 (not at all) to 5 (highly characteristic). Videos were coded by two raters from the local research team who were trained to reliability in the coding scheme by a gold standard NICHD rater (IRRs = 0.86–0.89 for full scheme). This approach to assessing and evaluating caregiving interactions has been demonstrated as both feasible and acceptable in India (Holla et al. 2021). All raters were blind to the depression and socio‐demographic status of the mothers.

#### 2.6.3 Covariates

Covariate data were obtained from a socio‐demographic questionnaire administered at baseline and included socio‐economic status (SES), maternal age, maternal education, parity and infant gender. Covariates were dichotomized where possible in order to simplify model interpretation and reduce the risk of over‐parameterization. A dichotomous variable was created to represent antenatal depression that merged data from each antenatal timepoint and represents caseness at any point. SES was derived from monthly family income and was not adjusted for family size. Mothers were classified as *Below Poverty Line/Low‐SES (<* *6000 INR per month), Higher Low‐SES (HLSES, 6000–11,999 INR per month), Middle‐SES (MSES, 12,000–30,999 per month)* or *Higher‐SES (HSES, ≥* *32,000 INR per month)*. These categories were informed by Indian government poverty line definitions relevant during the period of recruitment and data collection. Participants were classed as “Below Poverty Line” (BPL) if they were eligible for a BPL card. This was then collapsed into a dichotomous variable representing *BPL/HLSES (55.9%) (1)* versus *MSES/HSES (44.1%) (0)* for analyses. Maternal education was classified as *Up to Secondary (0)* versus *Above Secondary (1).* Parity was defined as whether the mother was *primiparous* or *multiparous* at the time of consent. Data regarding presence of an alternate caregiver (ACG) was obtained from a bespoke shared caregiving checklist. Data from T6 and T8 was combined to create a dichotomous variable that indicated whether there was an ACG *present* or *absent* at either postnatal timepoint.

#### 2.6.4 Infant Neurodevelopment

Infant cognitive and language development was assessed at 24 months using the BSID‐III (Bayley 2006). The BSID‐III is considered as a gold‐standard measure of infant development globally and has been widely used in low‐ and middle‐income settings (Breen et al. 2018). The tool measures development by direct observation across a number of domains, including *cognition, receptive language* and *expressive language.* These scales were administered in the local language and scored by research assistants who had been trained by a certified trainer. Further training and interrater reliability assessments were provided by a clinical psychologist with experience using the BSID‐III.

Raw scores for the cognitive, receptive language and expressive language subscales were converted into norm‐based scale scores (range 1–19, *M* = 10, SD = 3), and then into two composite scores (*M* = 100, SD = 15), one for the scaled cognitive score and one for the sum of the two language scaled scores. As no Indian norm scores for the BSID‐III exist, US‐based norms were used for the scoring, a practice that has been commonly adopted in the LMIC literature (Breen et al. 2018; Quevedo et al. 2012; Tran et al. 2013).

### 2.7 Analysis Plan

First, in order to create the chronic postnatal depression factor score, the tetrachoric correlations between the EPDS data at T5, T6, T8 and T9 were examined and the data were modeled as a single latent variable (Chronic Depression) using the gsem command in Stata version 14 (StataCorp 2015) and maximum likelihood estimation. A standardized factor score was then extracted (*M* = 0, SD = 1) and used for all subsequent analyses. This approach is similar to that taken by other key studies in this area (NICHD Early Child Care Research Network 1999; Mughal et al. 2023) and addresses challenges posed by the presence of unique thresholds for probably depression at different postnatal timepoints.

All subsequent analyses were conducted using Statistical Package for Social Sciences (SPSS) version 24 for Windows. Continuous variables were checked for normality and transformed where necessary. Following transformation, only chronic depression remained marginally skewed but was considered acceptable for use as a predictor in multiple linear regression.

Analyses were conducted independently for testing the associations between neurodevelopment and exposure to early postnatal and chronic depression and conducted using restricted samples consisting of participants who had complete data for all the included variables. Bivariate associations were examined using Pearson's correlations and, where appropriate, Spearman's or Tetrachoric correlations. *T*‐tests were also conducted to examine differences in outcomes by categorical predictors. Multiple linear regression models were constructed separately for each outcome, first in using data from the full sample, and then split by gender. Predictors were entered in a series of blocks, with each model adhering to the same basic structure. The covariates were entered in the first step, the main effects of depression and sensitivity to non‐distress were entered in the second step and the Maternal Depression × Sensitivity interaction terms were entered in the third step. All continuous variables were centered prior to creating the interaction terms. Interpretation of models will focus primarily on the covariate‐adjusted association between maternal depression, sensitivity to non‐distress, and infant neurodevelopment (cognition or language), as indicated in the hypotheses, rather than on the significance of the omnibus *F*‐test for the full model. This is a theory‐driven approach based on existing evidence that these three variables may be associated with each other. The influence of covariates on infant neurodevelopment will also be considered and discussed where statistically significant. Any results with regards to covariates should be considered as exploratory as they are not the primary focus of this paper. Overall model fit will be considered when interpreting the strength of any associations, including primary variables and covariates.

Participants were only included if they had full data on each relevant measure. The only exception to this was the maternal depression assessments. For the creation of the antenatal depression variable, in order to maximize data, depression was coded as present if it had been recorded at any time‐point in the antenatal period. Additionally, maximum likelihood estimation was used to account for any missing time‐points in the postnatal maternal depression assessments in the creation of the chronic depression factor.

A sensitivity power analysis was conducted using G*Power (version 3.1) based on a total sample size of 337. Assuming a two‐tailed *α* = 0.05 and 8–10 predictors/covariates in multiple linear regression models, the study had approximately 80% power to detect small‐to‐moderate main effects (Cohen's *f* ^2^ ∼ 0.02), corresponding to standardized regression coefficients of approximately *β* ∼ 0.14–0.15. These effect sizes are within the range typically reported in the literature examining associations between maternal depressive symptoms and maternal sensitivity (Madigan et al. 2019) with early child cognitive and language outcomes.

## 3 Results

### 3.1 Descriptive Statistics

At 8 weeks postpartum, using a cut‐off of ≥ 3 for postnatal depression, there was a prevalence of 21.7%. At 6, 12 and 24 months, using a cut off of ≥ 10, there was a prevalence of 14.9%, 16.9% and 14.9%, respectively. Table 2 shows descriptive statistics for key maternal exposure and child outcome variables.

**TABLE 2:** Key maternal exposure and child outcomes for the full study sample and split by sex.

Measure	Analyzed sample (*n* = 337)	Males (*n* = 174)	Females (*n* = 163)
Antenatal depression (depressed)	42.1%	42.5%	41.7%
T5 depression (depressed)	21.7%	23.6%	19.6%
Chronic depression (M, SD)	0.03 (0.98)	−0.11 (1.03)	0.05 (0.93)
Sensitivity to non‐distress (M, SD)	2.72 (0.95)	2.67 (0.89)	2.75 (1.01)
BSID cognitive (M, SD)	93.83 (8.23)	93.85 (8.63)	93.80 (7.81)
BSID language (M, SD)	97.75 (11.82)	96.24 (10.93)	99.36 (12.55)

Bivariate associations are presented in Table 3 for the final sample (i.e., participants with data for all variables), and in Table 4 for boys and girls separately. There was no significant association between postnatal or chronic depression and cognitive or language development. Chronic and postnatal depression were moderately to strongly positively associated with one another and with antenatal depression. Cognitive and language development showed a significant and strong association with one another, but cognitive development was not associated with any other covariates. There was a significant but weak association between language development and gender, indicating that girls showed higher language development than boys. Finally, maternal sensitivity to non‐distress demonstrated a significant but weak association with postnatal depression, indicating that mothers who reported higher depressive symptoms were likely to demonstrate lower levels of sensitivity.

**TABLE 3:** Bivariate associations between key study variables.

	Language	Postnatal depression	Chronic depression	Maternal sensitivity	Antenatal depression	Maternal age	Maternal education	Parity	Child sex	BW by GA^a^	ACG^b^	SES^c^
Cognitive	0.52**	−0.04	−0.03	0.03	−0.05	0.06	−0.02	−0.04	0.00	0.09	−0.03	0.07
Language		−0.10	−0.06	0.05	−0.07	0.04	0.01	−0.10	0.13*	0.04	−0.02	0.00
Postnatal depression			0.68**	−0.12*	0.51**	−0.05	−0.14	0.15	−0.05	0.01	−0.12*	−0.04
Chronic depression				−0.06	0.32**	−0.03	−0.09	0.01	−0.09	0.01	−0.12*	0.00
Maternal sensitivity					0.01	0.01	0.03	−0.04	0.03	−0.04	−0.03	−0.03
Antenatal depression						0.00	−0.07	−0.12	−0.01	−0.04	−0.02	−0.01
Maternal age							−0.04	0.36**	0.03	0.01	0.00	0.04
Maternal education								−0.15	−0.09	0.04	0.06	0.13
Parity									0.03	0.10	−0.06	−0.09
BW by GA											0.03	−0.03
ACG												0.07

*Note:* Tetrachoric correlations reported for associations between categorical predictors.

^a^ Birthweight by gestational age.

^b^ Alternate caregiver.

^c^ Socioeconomic status.

^*^ *p* < 0.05.

^**^ *p* < 0.001.

**TABLE 4:** Bivariate associations between key variables in chronic exposure sample separately for boys and girls.

	Cognitive	Language	Postnatal depression	Chronic depression	Maternal sensitivity	Antenatal depression	Maternal age	Maternal education	Parity	BW by GA^a^	ACG^b^	SES^c^
Cognitive		0.50**	−0.01	0.03	0.07	−0.01	0.21**	−0.02	0.07	−0.01	−0.14	0.20**
Language	0.55**		−0.09	−0.02	0.16*	−0.09	0.06	0.04	0.03	−0.05	−0.04	0.14
Postnatal depression	−0.06	−0.10		0.69**	−0.17	0.48**	−0.06	−0.15	0.17	0.04	−0.15*	−0.16
Chronic depression	−0.10	−0.07	0.67**		−0.07	0.29**	−0.05	−0.15*	−0.04	0.01	−0.12	−0.02
Maternal sensitivity	−0.02	−0.04	−0.07	−0.04		0.04	0.05	0.12	−0.02	−0.05	−0.01	−0.06
Antenatal depression	−0.11	−0.05	0.55**	0.35**	−0.01		−0.07	−0.08	−0.21*	0.06	0.00	−0.02
Maternal age	−0.12	0.00	−0.05	0.01	−0.04	0.08		−0.02	0.34**	0.04	−0.02	0.03
Maternal education	−0.01	0.00	−0.14	−0.04	−0.06	−0.04	−0.07		−0.03	−0.02	0.06	0.07
Parity	−0.17*	−0.22**	0.17	0.07	−0.06	−0.03	0.37**	−0.24*		0.08	−0.09	−0.18
BW by GA	0.20*	0.15	−0.04	−0.01	−0.02	−0.15	−0.01	0.09	0.12		0.06	0.03
ACG	0.20	0.15	−0.04	−0.01	−0.02	−0.15	−0.01	0.09	0.12	−0.01		0.10
SES	−0.09	−0.12	0.12	0.00	0.00	−0.03	0.06	0.19	−0.03	−0.10	0.03	

*Note:* Tetrachoric correlations reported for associations between categorical predictors. Boys in the top diagonal, girls in the bottom diagonal.

^a^ Birthweight by gestational age.

^b^ Alternate caregiver.

^c^ Socioeconomic status.

^*^ *p* < 0.05.

^**^ *p* < 0.001.

When bivariate associations were calculated for boys and girls separately, neither group showed any significant associations between chronic or postnatal depression and either cognitive or language development. Chronic, postnatal and antenatal depression were moderately to strongly positively associated with one another in mothers of both boys and girls. Language development demonstrated a positive but weak association with maternal sensitivity in boys but not girls, suggesting that more sensitive early caregiving was associated with better language outcomes in male infants. In boys, cognitive development was positively associated with maternal age and SES, indicating that boys with older mothers and those who were living in households with higher SES performed better. For girls, both cognitive and language development were negatively associated with parity, indicating that higher scores were associated with being the first‐born child. Cognitive development was also positively associated with birthweight by gestational age in girls, indicating that higher scores were associated with having a higher birthweight for their gestational age. Maternal sensitivity was not associated with either cognitive or language development in girls.

### 3.2 Multivariate Analysis

The effects of early postnatal depression and chronic depression on each outcome were investigated independently. Separate linear regression analyses were performed investigating each combination of exposure (postnatal or chronic) and outcome (cognitive or language) first in the whole sample (*n* = 337) and then by sex (male = 174, female = 163). Main effects were interpreted from the step the variables were entered in. Standardized *β* coefficients are reported to facilitate comparison of the relative strength of associations across predictors measured on different scales.

### 3.3 Early Postnatal Depression and Cognitive Development (Table 5)

#### 3.3.1 Full Sample

Early postnatal depression, maternal sensitivity to non‐distress, and the interaction between depression and sensitivity were not significantly associated with cognitive development. Overall, the model was non‐significant and accounted for 0.1% of the variance in cognitive development (*F*(11) = 0.80, *p* = 0.64).

#### 3.3.2 Boys

Early postnatal depression, maternal sensitivity to non‐distress, and the interaction between depression and sensitivity were not significantly associated with cognitive development in boys. Higher maternal age was associated with improved cognitive performance (*β* = 0.20, *p* = 0.01), as was higher SES (*β* = 0.21, *p* < 0.001) and the presence of an ACG was associated with poorer cognitive performance (*β* = −0.16, *p* = 0.05). Overall, the model was significant and accounted for 5.9% of the variance in cognitive development (*F*(10) = 2.01, *p* = 0.03).

#### 3.3.3 Girls

Early postnatal depression, maternal sensitivity to non‐distress, and the interaction between depression and sensitivity were not significantly associated with cognitive development in girls. Multiparity was associated with poorer cognitive performance (*β* = −0.18, *p* = 0.03) and higher birthweight by gestational age was associated with improved cognitive performance (*β* = 0.21, *p* = 0.01). Overall, the model was non‐significant and accounted for 4.5% of the variance in cognitive development (*F*(10) = 21.77, *p* = 0.07).

**TABLE 5:** Summary of linear regression models predicting cognitive development from early postnatal depression, sensitivity to non‐distress, and other covariates.

Cognitive development	Full sample (*n* = 337)				Boys (*n* = 174)				Girls (*n* = 163)			
	*b*	95% CI	*β*	*p*	*b*	95% CI	*β*	*p*	*b*	95% CI	*β*	*p*
Step 1												
Antenatal depression	−0.89	−2.68, 0.91	−0.05	0.33	0.31	−2.25, 2.87	0.02	0.81	−1.10	−3.52, 1.31	−0.07	0.37
Maternal age	0.16	−0.08, 0.4	0.08	0.18	0.44	0.1, 0.77	0.20	0.01	−0.09	−0.41, 0.22	−0.05	0.56
Maternal education	−0.55	−2.51, 1.41	−0.03	0.58	−0.68	−3.34, 1.98	−0.04	0.61	−0.94	−3.69, 1.8	−0.05	0.50
Parity	−1.39	−3.33, 0.56	−0.08	0.16	0.01	−2.72, 2.74	0.00	1.00	−2.86	−5.47, −0.24	−0.18	0.03
Birthweight by gestational age	63.48	−6.01, 132.97	0.10	0.07	−8.87	−108.24, 90.49	−0.01	0.86	126.68	33.08, 220.27	0.21	0.01
Child sex	0.14	−1.64, 1.92	0.01	0.88								
Alternate caregiver	−1.16	−4.03, 1.71	−0.04	0.43	−4.37	−8.41, −0.33	−0.16	0.03	2.56	−1.29, 6.41	0.10	0.19
SES	1.09	−0.7, 2.88	0.07	0.23	3.66	1.13, 6.19	0.21	< 0.001	−1.13	−3.52, 1.27	−0.07	0.35
Step 2												
Sensitivity to non‐distress	0.23	−0.71, 1.17	0.03	0.63	0.73	−0.72, 2.18	0.08	0.32	−0.21	−1.39, 0.97	−0.03	0.73
Postnatal depression	−0.23	−2.57, 2.12	−0.01	0.85	−0.17	−3.42, 3.07	−0.01	0.92	−0.15	−3.39, 3.08	−0.01	0.93
Step 3												
Postnatal depression × sensitivity to non‐distress	0.08	−2.22, 2.37	0.00	0.95	0.27	−3.14, 3.67	0.01	0.88	−0.41	−3.4, 2.58	−0.02	0.79

### 3.4 Early Postnatal Depression and Language Development (Table 6)

#### 3.4.1 Full Sample

Early postnatal depression, maternal sensitivity to non‐distress, and the interaction between depression and sensitivity were not significantly associated with language development. Multiparity was associated with poorer language development (*β* = −0.14, *p* = 0.02). Child sex was also associated with language development (*β* = 0.14, *p* = 0.01), indicating that girls performed better than boys. Overall, the model was non‐significant and accounted for 1.6% of the variance in language development (*F*(11) = 1.87, *p* = 0.13).

#### 3.4.2 Boys

Early postnatal depression was not significantly associated with language development in boys. Maternal sensitivity to non‐distress was associated with improved language development (*β* = 0.16, *p* = 0.04) but the interaction between postnatal depression and sensitivity was non‐significant. No other covariates were associated with language development. Overall, the model was non‐significant and accounted for 1.0% of the variance in language development (*F*(10) = 1.17, *p* = 0.31).

#### 3.4.3 Girls

Early postnatal depression, maternal sensitivity to non‐distress, and the interaction between depression and sensitivity were not significantly associated with language development in girls. Multiparity was associated with poorer language development (*β* = −0.29, *p* < 0.001) and birthweight by gestational age was associated with improved language development (*β* = −0.18, *p* = 0.03). Overall, the model was significant and accounted for 5.5% of the variance in language development (*F*(10) = 1.94, *p* = 0.04).

**TABLE 6:** Summary of linear regression models predicting language development from early postnatal depression, sensitivity to non‐distress, and other covariates.

Language development	Full sample (*n* = 337)				Boys (*n* = 174)				Girls (*n* = 163)			
	*b*	95% CI	*β*	*p*	*b*	95% CI	*β*	*p*	*b*	95% CI	*β*	*p*
Step 1												
Antenatal depression	−1.74	−4.29, 0.81	−0.07	0.18	−1.61	−4.98, 1.77	−0.07	0.35	−0.73	−4.6, 3.14	−0.03	0.71
Maternal age	0.24	−0.1, 0.58	0.08	0.16	0.15	−0.3, 0.59	0.05	0.51	0.38	−0.13, 0.88	0.12	0.14
Maternal education	0.19	−2.59, 2.98	0.01	0.89	0.54	−2.96, 4.05	0.02	0.76	−0.94	−5.33, 3.46	−0.03	0.68
Parity	−3.38	−6.14, −0.62	−0.14	0.02	0.1	−3.49, 3.7	0	0.95	−7.49	−11.69, −3.3	−0.29	< 0.001
Birthweight by gestational age	63.59	−35.29, 162.48	0.07	0.21	−37.4	−168.28, 93.48	−0.04	0.57	171.05	21.07, 321.04	0.18	0.03
Child sex	3.33	0.79, 5.87	0.14	0.01								
Alternate caregiver	−1.37	−5.45, 2.72	−0.04	0.51	−1.61	−6.93, 3.71	−0.05	0.55	−0.9	−7.06, 5.27	−0.02	0.77
SES	−0.02	−2.56, 2.53	0.00	0.99	3.09	−0.24, 6.42	0.14	0.07	−2.88	−6.72, 0.95	−0.11	0.14
Step 2												
Sensitivity to non‐distress	0.48	−0.86, 1.82	0.04	0.48	1.95	0.06, 3.84	0.16	0.04	−0.69	−2.57, 1.2	−0.06	0.47
Postnatal depression	−1.67	−5.00, 1.66	−0.06	0.32	−0.91	−5.13, 3.32	−0.04	0.67	−1.6	−6.77, 3.57	−0.05	0.54
Step 3												
Maternal depression × sensitivity to non‐distress	0.39	−2.87, 3.64	0.01	0.82	2.13	−2.3, 6.55	0.09	0.34	−1.23	−6.01, 3.54	−0.04	0.61

### 3.5 Chronic Depression and Cognitive Development (Table 7)

#### 3.5.1 Full Sample

Chronic depression, maternal sensitivity to non‐distress, and the interaction between depression and sensitivity were not significantly associated with cognitive development. None of the covariates were associated language development. Overall, the model was non‐significant and accounted for 0.1% of the variance in cognitive development (*F*(11) = 0.82, *p* = 0.61).

#### 3.5.2 Boys

Chronic depression, maternal sensitivity to non‐distress, and the interaction between depression and sensitivity were not significantly associated with cognitive development in boys. Higher maternal age was associated with improved cognitive performance (*β* = 0.20, *p* = 0.01), as was higher SES (*β* = 0.21, *p* < 0.001), and the presence of an ACG was associated with poorer cognitive performance (*B* = −0.16, *p* = 0.03). Overall, the model was significant and accounted for 5.9% of the variance in cognitive development (*F*(10) = 2.09, *p* = 0.03).

#### 3.5.3 Girls

Chronic depression, maternal sensitivity to non‐distress, and the interaction between depression and sensitivity were not significantly associated with cognitive development in girls. Multiparity was associated with poorer cognitive performance (*β* = −0.18, *p* = 0.03). Birthweight by gestational age was associated with improved cognitive performance (*β* = 0.21 *p* = 0.01). Overall, the model was significant and accounted for 6.4% of the variance in cognitive development (*F*(10) = 2.11, *p* = 0.03).

**TABLE 7:** Summary of linear regression models predicting cognitive development from chronic depression, sensitivity to non‐distress, and other covariates.

Cognitive development	Full sample (*n* = 337)				Boys (*n* = 174)				Girls (*n* = 163)			
	*b*	95% CI	*β*	*p*	*b*	95% CI	*β*	*p*	*b*	95% CI	*β*	*p*
Step 1												
Antenatal depression	−0.89	−2.68, 0.91	−0.05	0.33	0.31	−2.25, 2.87	0.02	0.81	−1.1	−3.52, 1.31	−0.07	0.37
Maternal age	0.16	−0.08, 0.40	−0.08	0.40	0.44	0.1, 0.77	0.2	0.01	−0.09	−0.41, 0.22	−0.05	0.56
Maternal education	−0.55	−2.51, 1.41	−0.08	0.58	−0.68	−3.34, 1.98	−0.04	0.61	−0.94	−3.69, 1.8	−0.05	0.50
Parity	−1.39	−3.33, 0.56	−0.08	0.16	0.01	−2.72, 2.74	0	1.00	−2.86	−5.47, −0.24	−0.18	0.03
Birthweight by gestational age	63.48	−6.01, 132.97	0.10	0.07	−8.87	−108.24, 90.49	−0.01	0.86	126.68	33.08, 220.27	0.21	0.01
Child sex	0.14	−0.165, 1.93	0.01	0.88								
Alternate caregiver	−1.16	−4.03, 1.71	−0.04	0.43	−4.37	−8.41, −0.33	−0.16	0.03	2.56	−1.29, 6.41	0.1	0.19
SES	1.09	−0.70, 2.88	0.01	0.23	3.66	1.13, 6.19	0.21	< 0.001	−1.13	−3.52, 1.27	−0.07	0.35
Step 2												
Sensitivity to non‐distress	0.23	−0.71, 1.70	0.03	0.63	0.76	−0.67, 2.19	0.08	0.29	−0.22	−1.4, 0.95	−0.03	0.71
Chronic depression	−0.16	−1.29, 0.97	−0.02	0.78	0.24	−1.31, 1.79	0.02	0.76	−0.6	−2.16, 0.95	−0.06	0.44
Step 3												
Chronic depression × sensitivity to non‐distress	0.29	−0.83, 1.41	0.03	0.61	−0.13	−1.83, 1.56	−0.01	0.88	1.16	−0.29, 2.6	0.12	0.12

### 3.6 Chronic Depression and Language Development (Table 8)

#### 3.6.1 Full Sample

Chronic depression, maternal sensitivity to non‐distress, and the interaction between depression and sensitivity were not significantly associated with language development. Multiparity was associated with poorer language development (*β* = −0.14, *p* = 0.02). Child sex was also associated with language development (*β* = 0.14, *p* = 0.01), indicating that girls performed better than boys. Overall, the model was non‐significant and accounted for 1.6% of the variance in language development (*F*(11) = 1.49, *p* = 0.14).

#### 3.6.2 Boys

Chronic depression was not significantly associated with language development in boys. Maternal sensitivity to non‐distress was associated with improved language development (*β* = 0.17, *p* = 0.03) but the interaction between postnatal depression and sensitivity was non‐significant. No other covariates were associated with language development. Overall, the model was non‐significant and accounted for 0.5% of the variance in language development (*F*(10) = 1.08, *p* = 0.38).

#### 3.6.3 Girls

Chronic depression, maternal sensitivity to non‐distress, and the interaction between depression and sensitivity were not significantly associated with language development in girls. Multiparity was associated with poorer cognitive performance (*β* = −0.29, *p* < 0.001) and birthweight by gestational age was associated with improved cognitive performance (*β* = −0.18, *p* = 0.03). Overall, the model was significant and accounted for 6.7% of the variance in language development (*F*(10) = 2.16, *p* = 0.02).

**TABLE 8:** Summary of linear regression models predicting language development from chronic depression, sensitivity to non‐distress, and other covariates.

Language development	Full sample (*n* = 337)				Boys (*n* = 174)				Girls (*n* = 163)			
	*b*	95% CI	*β*	*p*	*b*	95% CI	*β*	*p*	*b*	95% CI	*β*	*p*
Step 1												
Antenatal depression	−1.74	−4.29, 0.81	−0.07	0.18	−1.61	−4.98, 1.77	−0.07	0.35	−0.73	−4.6, 3.14	−0.03	0.71
Maternal age	0.24	−0.10, 0.58	0.08	0.17	0.15	−0.30, 0.59	0.05	0.51	0.38	−0.13, 0.88	0.12	0.14
Maternal education	0.19	−2.59, 2.98	0.01	0.89	0.54	−2.96, 4.05	0.02	0.76	−0.94	−5.33, 3.46	−0.03	0.68
Parity	−3.38	−6.14, −0.62	−0.14	0.02	0.10	−3.49, 3.7	0.00	0.95	−7.49	−11.69, −3.3	−0.29	< 0.001
Birthweight by gestational age	63.60	−35.29 162.48	0.08	0.14	−37.40	−168.28, 93.48	−0.04	0.57	171.05	21.07, 321.04	0.18	0.03
Child sex	3.33	0.79, 5.87	0.14	0.010								
Alternate caregiver	−1.37	−5.45, 2.72	−0.04	0.30	−1.61	−6.93, 3.71	−0.05	0.55	−0.9	−7.06, 5.27	−0.02	0.77
SES	−0.02	−2.56, 2.53	−0.001	0.990	3.09	−0.24, 6.42	0.14	0.07	−2.88	−6.72, 0.95	−0.11	0.14
Step 2												
Sensitivity to non‐distress	0.55	−0.78, 1.88	0.05	0.41	2.04	0.17, 3.90	0.17	0.03	−0.67	−2.55, 1.22	−0.05	0.49
Chronic depression	−0.31	−1.92, 1.28	−0.01	0.70	0.19	−1.84, 2.21	0.01	0.86	−0.79	−3.28, 1.69	−0.05	0.53
Step 3												
Chronic depression × sensitivity to non‐distress	0.62	−0.97, 2.22	0.05	0.44	−0.54	−2.74, 1.66	−0.04	0.63	1.73	−0.58, 4.04	0.11	0.14

## 4 Discussion

### 4.1 Summary of Main Findings

In the present study neither early postnatal nor chronic maternal depression were significantly associated with infant cognitive or language development. Maternal sensitivity to non‐distress did predict improved language outcomes in boys only, although this effect was small, and was not associated with cognitive outcomes. There was also no evidence that maternal sensitivity moderated the relationship between maternal depression and cognitive or language development, either in the full sample or the sex‐specific analyses. Some interesting sex‐specific findings did emerge regarding the covariates. Lower SES predicted poorer cognitive outcomes in boys only, while multiparity predicted poorer cognitive and language outcomes in girls only. Higher birthweight by gestational age, a proxy for nutrition, was associated with better cognitive and language outcomes in girls. Finally, the presence of an alternate caregiver was associated with significantly worse cognitive outcomes for boys, while for girls the association was positive, albeit not statistically significant. All effect sizes were small and should be interpreted with caution.

### 4.2 Comparison of Findings Regarding Early Postnatal Depression With HIC and LMIC Literature

The prevalence of depression in the current sample was highest at 8 weeks (21.7%) and decreased slightly but then remained fairly uniform from 6 to 24 months (14.9%–16.9%). These rates are similar to those found in a recent meta‐analysis of Indian studies (Upadhyay et al. 2017).

There have been inconsistent findings regarding the direct relationship between maternal postnatal depression and infant cognitive development in LMICs (Sridhar et al. 2025; Bluett‐Duncan et al. 2021). Thus, while the current finding from the multivariate analyses—that early postnatal and chronic depression were not significantly associated with infant neurodevelopment at 2 years—does not fully align with findings from HICs, it is not entirely incompatible with previous LMIC research. Specifically, within India, the literature is inconsistent (Koshy et al. 2021; Roy et al. 2022; Thomas et al. 2020; Patel et al. 2003). Interestingly, in a similar sample recruited from urban Vellore, poorer language, but not cognitive, development at 24 months was associated with maternal postnatal depression (Koshy et al. 2021). While we did not replicate this finding, the finding that poorer language development in boys was associated with poorer maternal SND may indicate that language development specifically is more vulnerable to early environmental risk factors.

In Barbados, Galler et al. (2000) found a significant association between self‐reported maternal depression at 7 weeks and cognitive development at 3 months, but not at 6 months. Each of the LMIC studies reviewed that did not find a significant association between infant neurodevelopment and postnatal depression assessed depression using a screening questionnaire and, with the exception of Tran et al. (2013), all assessed cognition at 12 months or later (Bluett‐Duncan et al. 2021). Conversely, a Brazilian study that did find a significant association between depression and language development at 12 months included women with a clinical diagnosis of depression only (Quevedo et al. 2012). It may be that mild to moderate postnatal depression can have a short‐term impact on development, but long‐term effects are restricted to children of mothers with more severe and enduring clinically diagnosed depression. Therefore, in the current study it is possible that any short‐term effect of early PND may have dissipated by 24 months and so was undetected.

There is a similar pattern in HIC research. Three out of four prospective studies found significant effects of postnatal depression on cognitive outcomes in the first 12 months. Two of these assessed depression using a clinical interview, with one finding an association at 12 months (Perra et al. 2015) while the other reported an association at 4 months that had dissipated by 13 months (Smith‐Nielsen et al. 2016). A third study found an effect of self‐reported depression on cognitive development at 12 months in very high‐risk sample (Lyons‐Ruth et al. 1986). Findings regarding the longer‐term effects were less consistent, although more studies reported a lack of significant association beyond 12 months (Kiernan and Huerta 2008; Piteo et al. 2012; Stein et al. 2008), than reported a significant association (Koutra et al. 2013; Kawai et al. 2017). Both studies which grouped mothers using a clinical diagnosis found a significant long‐term effect (Murray 1992; Conroy et al. 2012).

### 4.3 Caregiving and Infant Neurodevelopment in LMICs

This study reports evidence of a small but significant sex‐specific main effect of maternal sensitivity. There was a significant association between higher maternal SND and language development in boys, while the effect size was near zero and non‐significant for girls.

The association between maternal SND and language development is consistent with other HIC research. Leigh et al. (2011) reported that maternal sensitivity was associated improved vocabulary and expressive language outcomes at 15, 24 and 36 months (*n* = 1224). Effect sizes were all small but longitudinal associations tended to be stronger than concurrent associations. Another report (*n* = 1215) found that maternal sensitivity measured over multiple time‐points was a strong predictor of expressive language and verbal comprehension, over and above the contribution of maternal depression and other key covariates (NICHD Early Child Care Research Network 1999). Both papers indicate that consistency of maternal sensitivity over time is an important factor in predicting later language outcomes.

Consistent with prior evidence (Riva 2023) we found that female gender was associated with better language development. One possibility is that the maturational advantage held by girls means they are less vulnerable to environmental impacts on their outcomes. Sex‐specific vulnerabilities to compromised caregiving relationships (Murray et al. 1996, 2010) may stem from boys' comparatively lower self‐regulatory capacity, making them more reliant on caregiver scaffolding, while meta‐analytic evidence shows that girls display a significant advantage in effortful control, with stronger abilities to regulate and allocate attention and inhibit impulses than boys (Donald et al. 2019; Murray and Cooper 1997; Else‐Quest et al. 2006). Evidence indicates that such differences are not solely biologically determined but that early socialization processes, including differential caregiver responses to boys and girls from birth, play a significant role in shaping regulatory trajectories. For example, research demonstrates that caregivers tend to respond differently to male and female infants, reinforcing gender‐typed behaviors and shaping early emotional and attentional regulation patterns (Alexander and Wilcox 2012). It is likely, therefore that a complex interaction between biological predispositions and environmental input inclines male infants to be more vulnerable to the impact of impaired caregiving. Where male infants are unable to adequately self‐regulate and mothers are likewise unable to provide sufficient external regulation, they may miss out on key developmental opportunities. Our finding of a significant and positive association between maternal sensitivity and language development, unique to boys, is indicative of a consistent cross‐cultural effect whereby boys are more susceptible to changes in their environment during infancy.

There was no evidence in the current study that maternal sensitivity moderated the association between postnatal depression and infant neurodevelopmental outcomes, either in the full sample or in sex‐specific analyses.

There have been very few studies with this focus in LMIC settings. Black and et al. (2007) did find that caregiving was significantly and negatively associated with maternal depression in Bangladesh, and that it mediated the effect of a depression by infant temperament interaction on cognitive outcomes. Comparisons can also be drawn between the present study and a key HIC study which reported significant mediation and moderation effects (NICHD Early Child Care Research Network 1999). Here, caregiving was assessed and quantified across four time‐points in the first 3 years, as opposed to a single time point in this study. Thus, it is possible that long‐term impairments in maternal sensitivity are required to evoke changes in developmental outcomes (Leigh et al. 2011). They also observed a significant decline in sensitivity in depressed mothers across the second year and then some recovery in the third year. It is possible that the current study, which assessed caregiving at 6–12 months, did not capture the effects on sensitivity at a potentially difficult point of development, where infants exhibit behaviors which make interactions more challenging for depressed mothers.

It is also possible that certain socio‐cultural factors unique to India may have buffered infants against, or negated, the impact of maternal depression. Firstly, it is possible that the increased frequency and co‐occurrence of adversities in LMICs may be themselves affecting caregiving and development such that the impact of postnatal depression is negligible. Controlling for deprivation indicators within the current sample does not account for cross‐cultural disparities between HICs and LMICs and research has shown that differences in maternal sensitivity between ethnicities are most strongly predicted by differences in SES (Heng et al. 2018). These findings complement the family stress model which posits that socioeconomic disadvantage provokes an increase in familial stress which in turn disrupts sensitive caregiving, possibly by diverting maternal resources away from the child (Conger and Donnellan 2007). So, while there is evidence that maternal depression significantly affects caregiving behaviors that promote cognitive development in HIC settings, it is possible that it accounts for little unique variation in more adverse contexts.

Alternatively, it is possible certain socio‐cultural characteristics may make families in India more resilient to the impact of impaired caregiving. Chadda and Deb (2013) define India as a *collectivist* society in which families are far more involved in caring for their members than in traditionally individualistic societies. The difference between India and many HICs is perhaps best seen in the traditional joint‐family model found in India compared to the more common nuclear‐family model found in many HICs. Against this backdrop it is possible that the nurturing care provided within families and the emphasis on shared responsibility may buffer the child against the effects of impaired maternal sensitivity in the mother.

In this context, our finding that the presence of an ACG predicted poorer outcomes in boys, whereas there was a non‐significant trend in the opposite direction for girls, was unexpected. While this was a small effect and the limited nature of the measure that we used precludes firm conclusions, it is worth discussing briefly. There is evidence that male infants are more adversely affected by low‐quality non‐parental care (e.g., day‐care) than female infants (Brooks–Gunn et al. 2002). It is possible that ACGs, like day‐care providers, are unable to provide the same quality of scaffolding and regulation as mothers, leading to poorer developmental outcomes for male infants. Sex‐specific differences were also evident in the association between multiparity and both cognitive and language outcomes. In girls, there was a significant, but small, negative association between multiparity and neurodevelopment, while the standardized effect‐size for boys was near zero. Donald et al. (2019) similarly found primiparity predicted poorer language outcomes more strongly in girls. Lower birth order is linked to diluted parental resources and studies have now shown these effects apply to both biological and social ranks within the family (Kristensen and Bjerkedal 2007). Given the established presence of gender‐bias in favor of male infants in India (Barcellos et al. 2014), it is possible that a higher social rank given to a male infant lower‐down the biological birth‐order may reduce the impact of resource dilution on neurodevelopment.

### 4.4 Strengths and Limitations

The study was characterized by a number of design strengths. This was a prospective study of a large consecutive sample recruited from antenatal clinics serving a defined urban area in Bangalore, India. This allowed for the investigation of the prospective relationship between maternal depression and infant neurodevelopment. The version of the EPDS used in this study was subject to a rigorous, committee‐based translation process and validated in the local population against a psychiatric interview, with different cut‐off values established for the immediate perinatal period and the later postnatal period. This approach allowed for different response styles that were apparent at these different time‐points. The prospective design meant that EPDS data was available at three time points during pregnancy and four time points post‐birth, allowing for adequate control of antenatal depression and the creation of a relatively robust chronic depression factor across the first 2 years of infants' lives, in contrast with many other HIC and LMIC studies with only limited data collection during these timepoints. Additionally, neurodevelopmental outcomes and mother‐child interactions were independently observed and assessed by researchers using well established and widely used measures.

The BSID‐III is considered to be a gold‐standard measure of infant development and has been widely used at a global level, but there are no age‐specific normative data for Indian children. While the language score for the cohort aligns with expectations (*M* = 97.8), the mean cognitive score is substantially lower (*M* = 93.8) than the BSID‐III normative population. This aligns with results from another sample from a similar context in India (Koshy et al. 2021). That study demonstrated a widening gap in developmental scores from six to 36 months from HIC normative expectations, with language and cognitive scores at 24 months being 98.1 and 92.5, respectively. The similarity of these scores, and further investigation by Koshy et al. (2021), indicates that socioeconomic, home environment, and nutritional factors common to LMIC settings may contribute to this decline in neurodevelopmental scores relative to normative data generated from HIC samples.

Although the EPDS is widely used, it remains a screening instrument and does not provide a clinical diagnosis of depression. Nonetheless, while screening questionnaires like the EPDS may have detection limitations regarding severe clinical depression, they remain a highly feasible and meaningful measure for capturing the broader burden of maternal depressive symptoms. In resource‐constrained environments and large‐scale community cohorts where clinical diagnostic interviews are impractical, such screening tools are essential for identifying public health trends and preliminary risk factors.

Due to a delay in the release of funding, a number of infants were not able to complete the 6‐month assessment, meaning that some mother‐infant interactions were assessed at 6 months but others at 12 months. The two assessment periods were combined to maximize the sample size for analysis, but there may be small differences between parenting at 6 months and at 12 months that affected the results. However, both timepoints fall within the developmentally appropriate period for assessing maternal sensitivity, providing a theoretically coherent and statistically robust representation of infants' early caregiving experiences (NICHD Early Child Care Research Network 1999).

An additional limitation is that the measure used to index alternative caregiving was very brief. Mothers were asked whether or not there was anyone else who helped them take care of their child, and nearly 90% of mothers in the analysis sample endorsed this as yes. This likely reflects the timing of assessment, as typically Indian mothers return to live with their family for the first 6–9 months post‐birth, and so this may not be representative of the presence of an ACG throughout the first 2 years of the child's life. In addition, it is possible that the small negative association between the presence of an alternative caregiver and boys cognitive development may reflect some other characteristics of the 10% of mothers who reported that they did not have additional caregivers.

As stated above, a unique challenge in sample retention in this context is that it is common practice for the women in the cohort to migrate to their maternal home toward the end of pregnancy and to remain there for the first 6–9 months postpartum. Whenever possible, research assistants traveled to these homes to complete assessments, but in many instances the participants had moved too far from the research site and so could not be followed up at that time.

Finally, although the present study included a relatively large sample for a longitudinal cohort in a low‐ and middle‐income country (*N* = 337), statistical power should be considered when interpreting null findings. With this sample size, the analyses were adequately powered to detect small‐to‐moderate main effects in the full sample, which are typical of associations reported between maternal postnatal depression and sensitivity and early child cognitive and language outcomes. However, sex‐stratified analyses and tests of interaction effects require larger samples. In addition, while there were differences in effect‐size and effect‐direction for certain variables in the sex‐stratified analyses, these were small and we did not formally test for the statistical significance of these differences. We also did not correct for multiple comparisons. As such, null findings for moderation and sex‐specific effects should be interpreted cautiously.

### 4.5 Implications

A key finding from this study is that mild‐moderate self‐reported early postnatal and chronic depression is not associated with impaired cognitive or language development in infants at 2 years in a low‐income urban community sample in South India before or after adjusting for covariates. This has potential positive implications for child development in resource constrained settings. Maternal postnatal depression is relatively common and so the indication that it is not impacting on later infant cognitive development may be interpreted positively. However, a degree of caution is required in interpreting this finding as the limited research carried out regarding this association within India is inconsistent, both in methodology and results.

There have been consistent findings in both HIC and LMIC settings that provide evidence of a significant effect of more severe, clinically diagnosed depression. This finding should not, therefore, be interpreted as meaning that postnatal depression does not represent a risk for child development. Instead, it is important for future studies to investigate the impact of clinically diagnosed postnatal depression on child outcomes in a community setting. For clinicians, this finding implies that while screening tools may be useful for identifying at‐risk mothers, further assessment to determine the presence of clinical diagnosis will be useful in delineating those mothers and infants who are most at risk. This is an important implication in LMIC settings as it means that the limited resource available can be more strategically allocated.

Maternal sensitivity was found to promote language development in male infants. This is consistent with established HIC research and implies that boys are more reliant on the regulatory scaffolding provided by a sensitive and responsive caregiver. Targeted interventions to promote optimal child development for male infants and their mothers in contexts where the caregiving relationship has been significantly compromised are therefore likely to provide a substantial return on investment.

It will be important for future studies to look to disentangle the roles of sociocultural factors and factors associated with lower socioeconomic status in order to better understand the dynamic of the relationship between maternal mental health, caregiving quality, and infant development.

### 4.6 Conclusion

This is only the second study to explore the prospective relationship between early postnatal depression and infant cognitive development in India, and the first to explore the impact of chronic depression and the interplay with maternal sensitivity. Adjusted estimates showed no association between early postnatal or chronic exposure to maternal depression and impaired cognitive or language outcomes. There was also no evidence that maternal sensitivity moderated this association, but maternal sensitivity did significantly predict language development in boys. The effect was small and should be interpreted with caution. However, the findings extend previous evidence from HICs that boys may be more reliant on the external regulation provided by mothers for optimal development to a LMIC setting.

## Author Contributions

**Matthew Bluett‐Duncan:** investigation, methodology, writing – review and editing, formal analysis, data curation, writing – original draft. **Thomas Kishore:** conceptualization, writing – review and editing, supervision, investigation. **Veena Satyanarayana:** writing – review and editing, supervision, investigation. **Supraja TA:** investigation, writing – review and editing, methodology, project administration, data curation. **Laura Bozicevic:** writing – review and editing, project administration, investigation. **Chaithra Holla:** writing – review and editing, project administration, data curation, investigation. **Nicky Wright:** writing – review and editing, formal analysis, methodology, investigation. **Ellie Preece:** writing – review and editing, resources. **Andrew Pickles:** conceptualization, funding acquisition, writing – review and editing, supervision. **Prabha Chandra:** conceptualization, investigation, funding acquisition, writing – review and editing, project administration, supervision. **Helen Sharp:** conceptualization, investigation, funding acquisition, writing – review and editing, project administration, supervision.

## Ethics Statement

Ethical approval for phases 1 to 5 of Bangalore Child Health and Development Study was granted by the National Institute of Mental Health and Neurosciences (NIMHANS) Institutional Ethics Committee (IEC) on 22nd June 2013. These phases were solely funded by the Indian Council of Medical Research and so did not require UK ethics approval. Ethical approval for phases 6–9 was granted by the NIMHANS IEC on 2nd July 2015 and by the University of Liverpool ethics committee on 1st March 2016. All research was conducted in compliance with the American Psychological Association ethical standards for the treatment of human participants. Participants gave written informed consent for data collection at multiple phases within the study for themselves and their children.

## Conflicts of Interest

The authors declare no conflicts of interest.

## Supporting information


Supporting Information S1

**Figure S1:** EPDS receiver‐operating characteristic (ROC) curve for the EPDS predicting clinically diagnosed depression during the antenatal period, sensitivity versus 1‐specificity. Area under the curve = 0.734.

**Figure S2:** Receiver‐operating characteristic (ROC) curve for the EPDS predicting clinically diagnosed depression during the postnatal period, sensitivity versus 1‐specificity. Area under the curve = 0.95.

**Table S1:** Number of participants with and without data for included variables.

## Data Availability

The data that support the findings of this study are available from the corresponding author upon reasonable request.
