# Supplementary material for: Early and Chronic Postnatal Depression, Maternal Sensitivity to Non‐Distress and Infant Neurodevelopmental Outcomes in an Indian Birth Cohort
Source: Infancy. 2026 Jun 25;31(4):e70103. doi: 10.1111/infa.70103 (PMC13305149; doi:10.1111/infa.70103)
Supplement: Supplementary file 1 — Supporting Information S1 [file INFA-31-0-s001.docx]

**Appendix 1: EPDS Validation Study**

***Summary***

The objective of this study was to assess the validity of a Kannada version of the Edinburgh Postnatal Depression Scale (EPDS) and to establish separate cut-off points for probable depression in the antenatal and postnatal periods. Separate samples of 150 pregnant women and 98 mothers of young infants completed the EPDS and a short demographic interview. The presence or absence of a clinical diagnosis of a major depressive episode was confirmed by the Mini International Neuropsychiatric Interview (MINI), administered by a psychiatrist or psychiatric social worker blind to the EPDS response. The ROC curve analysis revealed that the EPDS was a fair quality instrument during the antenatal period (AUC = .734) and a score of 3 or more was selected as the optimal cut-off to screen for probable antenatal depression in women (sensitivity = 62.5%, specificity = 73.9%). Due to patterns of reporting in the BCHADS sample, this cut-off was also applied to the 8-week postnatal assessment. The ROC curve analysis during the postnatal period indicated that the EPDS is an excellent quality instrument at this time-point (AUC = 0.945) and a score of 10 or more was selected as the optimal cut-off to screen for probable postnatal depression (sensitivity = 95.2%, specificity = 83.1%). This cut-off was applied to the 6, 12 and 24 month assessments in BCHADS.

***1. Introduction***

A recent meta-analysis of postnatal depression prevalence showed that 29 out of 38 studies conducted in India used the Edinburgh Postnatal Depression Scale (EPDS) (Cox, Holden, & Sagovsky, 1987), either independently or alongside another measure. However, only 8 of these studies reported a validated cut-off point for probably depression while the majority utilised thresholds validated in western populations. Evidence suggests that the EPDS functions differently in different settings, with thresholds especially likely to vary between different cultures (Gibson, McKenzie-McHarg, Shakespeare, Price, & Gray, 2009). Therefore, it is important to validate each adaptation within its context.

The EPDS has previously been translated into Kannada and validated in a rural population (Fernandes et al., 2011). The authors report a cut-off score of 13 or more (sensitivity=100%, specificity=84.90%, AUC=0.95) for detecting prenatal depression but note the findings may not be generalisable to an urban setting where different cut-offs may be expected. In view of this, the current study recruited two independent samples, one antenatal and one postnatal, to validate a Kannada version of the EPDS in an urban setting.

***2. Method***

This study used a cross-sectional design and was carried out between September 2015 and December 2015 (antenatal sample) and from August 2019 to October 2019 (postnatal sample).

*2.1. Antenatal Sample*

A consecutive sample of 150 women were recruited from the antenatal clinic at the Banashankari Urban Primary Health Centre in Bangalore. Pregnant women living in low income areas of urban Bangalore (India) and registered with the Antenatal clinic at a Government Referral Hospital (GRH) in South Bangalore were potentially eligible to participate. Women who had a major mental illness such as psychosis or a bipolar disorder, who were identified to have major health complications during the current pregnancy or were currently using alcohol or other psychoactive substances were excluded.

*2.2. Postnatal Sample*

A consecutive sample of 98 mothers were recruited from an antenatal/maternity clinic in South Bangalore when attending routine immunisation appointments with their infants. Mothers living in low income areas of urban Bangalore (India) and registered with the Antenatal clinic at a Government Referral Hospital (GRH) in South Bangalore were potentially eligible to participate. Women who had a major mental illness such as psychosis or a bipolar disorder, who were identified to have major health complications during the current pregnancy or were currently using alcohol or other psychoactive substances were excluded.

*2.3. Measures*

*Edinburgh Postnatal Depression Scale (EPDS):* The EPDS is a 10-item Likert scale self-report instrument designed to detect postnatal depression by focusing on the cognitive and affective aspects of depression rather than somatic symptoms. Each item addresses a distinct symptom of postnatal depression and is rated using a unique set of response items, scored 0-3. The instrument gives a total score of 0-30, with a higher score indicating greater distress.

The EPDS was translated into the local language (Kannada) following World Health Organisation guidelines for measure translation. Forward translation was done by a researcher from the team who was a native speaker of Kannada with bilingual proficiency in Kannada and English. The translated version was then reviewed by experts in the field to ensure the appropriateness of the terms and phrases used in the questions and revisions were made based on their suggestion. This version was then back-translated to English by a translator with bilingual proficiency. The original English questionnaire, the translation in Kannada and the back-translated version were compared and reviewed to identify any differences and appropriate modifications were made. Finally, a pilot study was conducted with 10 pregnant mothers from the target population. Final modifications were made to the phrasing of items 1, 2, 4, 5 and 6 based on pilot feedback.

*Mini International Neuropsychiatric Interview* *(MINI):* The reference standard diagnostic interview was the MINI version 5.0.0 (Lecrubier et al., 1997), a short structured diagnostic interview for DSM-IV and ICD-10 psychiatric disorders. The MINI has been validated against the SCID for DSM-III R and the CIDI for ICD-10 (Sheehan, et al. 1997; Spies et al. 2009), and has been used extensively in field research in India (Fernandes et al., 2011).

*2.4. Procedure*

In both studies the women first completed the EPDS and a short demographic interview. Although the EPDS was originally designed to be self-administered, low literacy levels in the sample meant that the scale had to be researcher administered, with researchers reading out each item and then the set of responses to be selected by participants. The MINI interview was then conducted in a separate room by either a psychiatrist or psychiatric social worker who was blind to the responses given on the EPDS.

*2.5. Analysis*

All analyses were carried out using Statistical Package for Social Sciences (SPSS) version 24 for Windows. Descriptive statistics regarding the sociodemographic characteristics and mean EPDS scores were produced first and are presented alongside corresponding statistics from the main BCHADS sample. The sample was categorised into cases and non-cases based on a MINI diagnosis of major depressive disorder (MDD). Receiver-operating characteristic curves were then calculated to determine sensitivity, specificity, and the area under the curve (AUCs).

***3. Results***

*3.1. Sample Characteristics*

Sample demographics for the antenatal and postnatal validation cohorts are presented alongside the baseline demographics for the BCHADS cohort in table A1.

Table A1: Comparison of sample demographics

| **Characteristic** | | **Antenatal Validation** | **Postnatal Validation** | **BCHADS Baseline** |
| --- | --- | --- | --- | --- |
| **Maternal Age** | | 22.77 (3.21) | 26.52 (4.60) | 22.92 (3.66) |
| **Maternal Education** (years) | | 9.89 (2.76) | 11.16 (3.53) | 9.81 (2.60) |
| **Occupation** (Employed) | | 7.3% | 9.2% | 13.8% |
| **SES** | BPL/LSES | 8.8% | 5.1% | 11.3% |
|  | Upper-LSES | 52.7% | 26.5% | 44.8% |
|  | MSES | 38.5% | 51% | 41.2% |
|  | USES | 0.0% | 14.3% | 2.8% |
| **Religion** | Hindu | 78.0% | 67.3% | 83.5% |
|  | Muslim | 21.3% | 26.5% | 15.4% |
|  | Other | 0.7% | 6.1% | 1.1% |

*3.2. Depression Prevalence*

The mean EPDS score during the antenatal period was 2.93 (SD = 5.32). 16 women met diagnostic criteria for a major depressive episode on the MINI, giving an antenatal prevalence of 10.6%.

The mean EPDS score during the postnatal period was 7.61 (SD = 5.37). 21 women met diagnostic criteria for a major depressive episode on the MINI, giving a postnatal prevalence of 21.4%.

The current mean EPDS scores are presented alongside mean EPDS scores from each BCHADS study phase in table A2. Due to similarities in mean scores between the antenatal and early postnatal (T5 – 8 weeks) phase in BCHADS and then the sharp increase in mean scores from 6 months postnatal onwards, it was decided to apply the threshold validated in the antenatal period to the 8 week BCHADS assessments. As a result, the present study validates two cut-offs, one for the immediate perinatal period, and one for the later postnatal period going through into infancy.

Table A2: Summary of EPDS scores showing means, SDs, and number of cases.

| **Assessment** | **N** | **Mean (SD)** | **Total Cases** |
| --- | --- | --- | --- |
| **Cut-off = ≥3** | | | |
| Antenatal Validation | 150 | 2.93 (5.32) | 45 (30.0%) |
| T1 – 1^st^ trimester | 695 | 2.34 (5.10) | 168 (24.2%) |
| T2 – 2^nd^ trimester | 698 | 2.30 (4.56) | 177 (25.4%) |
| T3 – 3^rd^ trimester | 607 | 1.73 (4.09) | 110 (18.1%) |
| T5 – 8 weeks postnatal | 545 | 1.79 (4.28) | 105 (19.3%) |
| **Cut-off = ≥10** | | | |
| Postnatal Validation | 98 | 7.61 (5.37) | 33 (33.7%) |
| T6 – 6 months postnatal | 415 | 4.34 (5.75) | 64 (15.4%) |
| T8 – 12 months postnatal | 549 | 4.20 (5.39) | 87 (15.8%) |
| T9 – 24 months postnatal | 674 | 4.17 (5.09) | 97 (14.4%) |

*3.3. Antenatal AUC/ROC Curve Analysis*

The receiver operating characteristic (ROC) curve comparing the antenatal EPDS to the MINI diagnosis of depression is presented in supplementary figure 1. The resulting area under the curve indicated that the EPDS is a fair quality instrument at this time-point (AUC = 0.734, 95% CI: 0.64, 0.80). A score of 3 or more was selected as the optimal cut-off to screen for probable perinatal depression in women (sensitivity = 62.5%, specificity = 73.9%).

*3.4. Postnatal AUC/ROC Curve Analysis*

The ROC curve comparing the postnatal EPDS to the MINI diagnosis of depression is presented in supplementary figure 2. The resulting area under the curve indicated that the EPDS is an excellent quality instrument at this time-point (AUC = 0.945, 95%: 0.88, 0.98). A score of 10 or more was selected as the optimal cut-off to screen for probable perinatal depression in women (sensitivity = 95.2%, specificity = 83.1%).

***4. Summary of Findings***

Cut-offs for detecting probable maternal depression using a Kannada version of the EPDS were validated in the independent antenatal and postnatal samples.

*4.1. Antenatal Cut-Off*

Results from the antenatal sample indicated that the EPDS is fair quality instrument in this period and a cut-off of 3 or more was selected for detecting probable depression (sensitivity = 62.5%, specificity = 73.9%). Mean EPDS scores during this period were quite low and a similar pattern was present in the antenatal and immediate postnatal (8 weeks) periods in the main BCHADS sample. Therefore the antenatal cut-off will be applied to the immediate perinatal period, including assessments at each trimester of pregnancy and at 8 weeks postnatal.

Using this threshold, if you surveyed 100 women in the general population (prevalence 20%), 12.5 of the 20 women with depression would be correctly identified as depressed and 20.9 women incorrectly identified as depressed. If you surveyed 100 women in a clinic sample (prevalence 50%) 31.3 of the 50 women would be correctly identified as depressed and 14.7 out of 50 women would be incorrectly identified as depressed.

*4.2 Postnatal Cut-off*

Results from the postnatal sample indicated that the EPDS is an excellent quality instrument in this period and a cut-off of 10 or more was selected for detecting probable depression (sensitivity = 95.2%, specificity = 83.1%). This cut-off will be applied to the late postnatal period, including assessments at 6, 12 and 24 months.

Using this threshold, if you surveyed 100 women in the general population (prevalence 20%), 19.0 of the 20 women with depression would be correctly identified as depressed and 13.5 women incorrectly identified as depressed. If you surveyed 100 women in a clinic sample (prevalence 50%) 47.6 of the 50 women would be correctly identified as depressed and 8.5 out of 50 women would be incorrectly identified as depressed.
