# Supplementary material for: Early and Chronic Postnatal Depression, Maternal Sensitivity to Non‐Distress and Infant Neurodevelopmental Outcomes in an Indian Birth Cohort
Source: Infancy. 2026 Jun 25;31(4):e70103. doi: 10.1111/infa.70103 (PMC13305149; doi:10.1111/infa.70103)
Supplement: Supplementary file 2 — Figure S1: EPDS receiver‐operating characteristic (ROC) curve for the EPDS predicting clinically diagnosed depression during the antenatal period, sensitivity versus 1‐specificity. Area under the curve = 0.734. [file INFA-31-0-s002.docx]

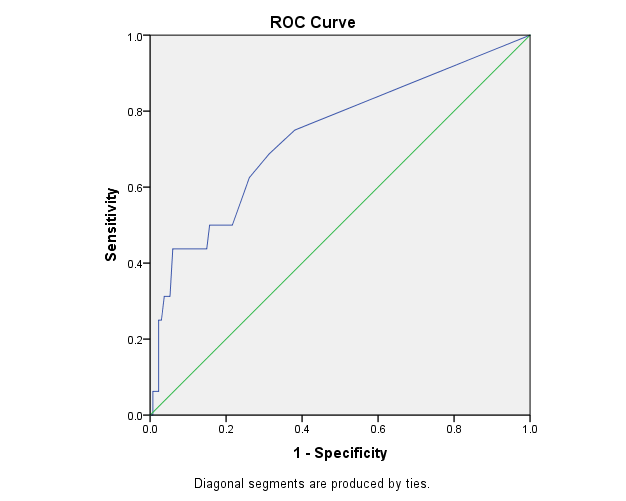


Supplementary Figure 1: EPDS Receiver-operating characteristic (ROC) curve for the EPDS predicting clinically diagnosed depression during the antenatal period, sensitivity vs. 1-specificity. Area under the curve=0.734.
