# Supplementary material for: Early and Chronic Postnatal Depression, Maternal Sensitivity to Non‐Distress and Infant Neurodevelopmental Outcomes in an Indian Birth Cohort
Source: Infancy. 2026 Jun 25;31(4):e70103. doi: 10.1111/infa.70103 (PMC13305149; doi:10.1111/infa.70103)
Supplement: Supplementary file 3 — Figure S2: Receiver‐operating characteristic (ROC) curve for the EPDS predicting clinically diagnosed depression during the postnatal period, sensitivity versus 1‐specificity. Area under the curve = 0.95. [file INFA-31-0-s004.docx]

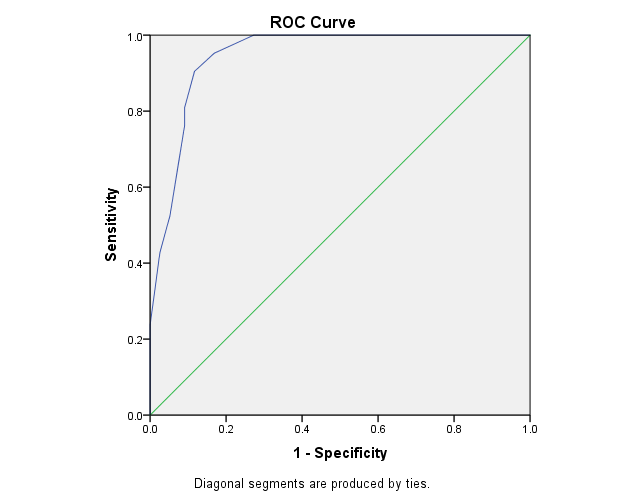


Supplementary Figure 2: Receiver-operating characteristic (ROC) curve for the EPDS predicting clinically diagnosed depression during the postnatal period, sensitivity vs. 1-specificity. Area under the curve=0.95.
