# Supplementary material for: Early and Chronic Postnatal Depression, Maternal Sensitivity to Non‐Distress and Infant Neurodevelopmental Outcomes in an Indian Birth Cohort
Source: Infancy. 2026 Jun 25;31(4):e70103. doi: 10.1111/infa.70103 (PMC13305149; doi:10.1111/infa.70103)
Supplement: Supplementary file 4 — Table S1: Number of participants with and without data for included variables. [file INFA-31-0-s003.docx]

Supplementary Table – Number of participants with and without data for included variables

| **Variables** | Data Present | Data Missing |
| --- | --- | --- |
| Antenatal Depression | 741 | 1 |
| Maternal Age | 740 | 0 |
| Maternal Education | 740 | 2 |
| Parity | 739 | 3 |
| Birthweight by Gestational Age | 680 | 62 |
| Child Sex | 741 | 1 |
| Alternate Caregiver | 587 | 155 |
| SES | 666 | 76 |
| Sensitivity to Non-Distress | 513 | 229 |
| Chronic Depression (at least 2 timepoints) | 741 | 1 |
| Early Postnatal Depression (T5) | 538 | 204 |
